# Preoperative Frailty-Inflammation Phenotypes and Early Functional and Patient-Reported Recovery After Head-and-Neck Oncologic Reconstruction: An Exploratory Prospective Single-Center Cohort Study

**DOI:** 10.3390/jcm15187134

**Published:** 2026-09-14

**Authors:** Sonia Roxana Burtic, Bogdan Florin Capastraru, Panche Taskov, Tudorel Mihoc, Codrina Mihaela Levai, Andreea Mihaela Kis, Roxana Manuela Fericean, Mihaela-Iuliana Ciortan (Sirbu), Daniel-Laurentiu Pop, Adelina Maria Jianu

**Affiliations:** 1 Doctoral School, “Victor Babes” University of Medicine and Pharmacy, Eftimie Murgu Square 2, 300041 Timisoara, Romania; dr.soniaburtic@umft.ro (S.R.B.); mihaela.sirbu@umft.ro (M.-I.C.); daniel.pop@umft.ro (D.-L.P.); 2 Discipline of Medical Communications, Department II Microscopic Morphology, “Victor Babes” University of Medicine and Pharmacy, Eftimie Murgu Square 2, 300041 Timisoara, Romania; codrinalevai@umft.ro; 3 Research Center for Medical Communication, “Victor Babes” University of Medicine and Pharmacy, Eftimie Murgu Square 2, 300041 Timisoara, Romania; 4 Department XV, Disciplina Ortopedie-Traumatologie I, “Victor Babes” University of Medicine and Pharmacy, Eftimie Murgu Square 2, 300041 Timisoara, Romania; bogdan.capastraru@umft.ro; 5 Discipline of Plastic and Reconstructive Surgery, Faculty of Medicine, “Victor Babes” University of Medicine and Pharmacy, Eftimie Murgu Square 2, 300041 Timisoara, Romania; panche.taskov@umft.ro; 6 Department X, General Surgery II, Discipline of Surgical Emergencies, “Victor Babes” University of Medicine and Pharmacy, Eftimie Murgu Square 2, 300041 Timisoara, Romania; mihoc.tudorel@umft.ro; 7 Department I—Management and Communication, Faculty of Dental Medicine, “Victor Babes” University of Medicine and Pharmacy, Eftimie Murgu Square 2, 300041 Timisoara, Romania; 8 Discipline of Dermatology, Faculty of Medicine, “Victor Babes” University of Medicine and Pharmacy, Eftimie Murgu Square 2, 300041 Timisoara, Romania; 9 Center for the Morphologic Study of the Skin (MORPHODERM), “Victor Babes” University of Medicine and Pharmacy, Eftimie Murgu Square 2, 300041 Timisoara, Romania; 10 Otorhinolaryngology (ENT) Department, “Victor Babes” University of Medicine and Pharmacy, Eftimie Murgu Square 2, 300041 Timisoara, Romania; 11 Department of Anatomy and Embryology, Faculty of Medicine, “Victor Babes” University of Medicine and Pharmacy, Eftimie Murgu Square 2, 300041 Timisoara, Romania; adelina.jianu@umft.ro

**Keywords:** head and neck neoplasms, reconstructive surgical procedures, frailty, inflammation, deglutition disorders

## Abstract

**Background/Objectives:** Early recovery after major head-and-neck reconstruction is not determined by flap survival alone. This exploratory study examined whether preoperative frailty-inflammatory phenotypes are associated with delayed swallowing recovery, persistent nutritional dependence, and poorer quality of life at 12 weeks. **Methods:** In a prospective single-center cohort study, 84 adults undergoing major head-and-neck oncologic reconstruction were assigned to three clinically defined phenotypes: low-risk (*n* = 28), intermediate (*n* = 29), and combined frailty-inflammatory (*n* = 27). Twelve-week outcomes included the Functional Oral Intake Scale (FOIS), MDADI, EORTC global health, PEG dependence, and a composite poor-recovery endpoint. Because of the small sample size, all estimates are reported to two significant figures, and every analysis is regarded as hypothesis-generating. **Results:** Compared with the low-risk group, the combined phenotype was older (63 ± 9 vs. 55 ± 9 years) and had a higher mFI-5 (3.2 ± 0.6 vs. 1.0 ± 0.7) and CRP/albumin ratio (2.2 ± 0.6 vs. 0.6 ± 0.2; all *p* < 0.001). At 12 weeks the combined phenotype showed greater weight loss (12 ± 2%), lower FOIS (3.2 ± 1.3), lower MDADI (51 ± 7), and PEG dependence in 52% (all *p* ≤ 0.002). A higher mFI-5 was associated with poor recovery (adjusted OR 2.1 per point; 95% CI 1.0–4.1; *p* = 0.04), an estimate whose confidence interval is wide and imprecise. **Conclusions:** In this small cohort a frailty-inflammatory framework was associated with clinically meaningful heterogeneity in early recovery. The findings are preliminary and require prospective, adequately powered, multicenter validation before any clinical use.

## 1. Introduction

Major head-and-neck oncologic reconstruction has traditionally been judged by technical endpoints such as flap viability, wound closure, and avoidance of catastrophic failure [1,2]. These endpoints are no longer sufficient: patients and multidisciplinary teams increasingly define success by how quickly oral intake, communication, symptom control, and social reintegration are restored [3]. Anatomical repair and lived functional recovery may therefore diverge during the first postoperative months.

The early postoperative interval is decisive because it concentrates the competing demands of wound healing, nutritional adequacy, symptom control, rehabilitation adherence, and adjuvant oncologic planning [1,3]. A technically successful reconstruction can still be followed by persistent dysphagia, accelerating weight loss, and psychological distress. Studies focused exclusively on complications therefore miss an important layer of variance in patient-centered recovery [2,4].

Frailty offers one explanatory layer because it reflects vulnerability beyond chronological age [4,5]. In surgical populations it captures diminished physiologic reserve, comorbidity burden, and limited resilience to catabolic stress [5,6]. Its relevance is amplified in head-and-neck surgery, where recovery depends on coordinated performance across nutrition, mobility, airway care, and communication [6,7]; even modest baseline frailty may magnify the impact of a technically uncomplicated operation [8].

Inflammatory and nutritional biomarkers provide a complementary perspective [9,10]. The CRP/albumin ratio combines systemic inflammatory burden with protein reserve, while albumin and skeletal muscle indices approximate nutritional resilience [11,12]. These variables are available before surgery and are simple to interpret [9,13]. Isolated biomarkers, however, may be less informative than clinically interpretable phenotypes that integrate inflammation with whole-patient vulnerability [14].

Flap healing depends on local determinants (recipient-bed vascularity, defect geometry, prior irradiation, hematoma, and microbial contamination) and on general determinants (age, nutritional status, diabetes, smoking, anemia, and systemic inflammatory burden) [15,16]. Pro-inflammatory cytokines impair endothelial function, promote microthrombosis, and disturb the transition from the inflammatory to the proliferative phase of wound healing [17], while sustained inflammation depletes the albumin and amino-acid pools required for collagen synthesis [11,17].

Postoperative swallowing and quality-of-life outcomes are multidimensional [18]. FOIS reflects graded oral intake, MDADI captures dysphagia-specific burden, EORTC global health reflects broader health perception, H&N35 swallowing estimates symptom severity, and HADS tracks emotional strain. Examined together, these domains help explain why patients undergoing similar operations follow very different recovery paths [3,18].

This is an exploratory, hypothesis-generating analysis of a prospective 12-week cohort at a single university-affiliated reconstructive service. Its objectives were to describe the baseline risk structure, perioperative course, and 12-week functional and patient-reported outcomes of three preoperative frailty-inflammatory phenotypes, and to examine, using a deliberately compact set of models, whether these phenotypes are associated with early recovery. Given the modest cohort size, no attempt is made to establish predictive validity.

## 2. Materials and Methods

### 2.1. Study Design and Ethical Framework

This prospective cohort study was conducted at the tertiary reconstructive head-and-neck practice affiliated with the “Victor Babeș” University of Medicine and Pharmacy, Timișoara, Romania. The protocol was approved by the Institutional Ethics Committee in accordance with the Declaration of Helsinki. Informed consent was obtained from all participants before study participation. The study window comprised the preoperative period, the index hospitalization, and a standardized 12-week follow-up.

Participants were prospectively enrolled from March 2024 to October 2025. Clinical and perioperative data were prospectively collected from electronic medical records, operative and anesthesia logs, ICU and ward documentation, outpatient rehabilitation notes, and validated questionnaires administered preoperatively and at 12 weeks. Variables were double-entered on a pre-specified case report form and cross-checked by two investigators (S.R.B. and B.F.C.); patient-level data were de-identified before analysis.

Eligibility criteria were defined a priori. Patients were included if they were aged 18 years or older, had a histologically confirmed head-and-neck malignancy, underwent primary or salvage resection requiring free-flap or pedicled-flap reconstruction, had preoperative C-reactive protein, serum albumin, and complete blood count obtained within 14 days of surgery, and had baseline FOIS and MDADI scores. Patients were excluded for palliative-intent or non-oncologic surgery, uncontrolled infection or sepsis, decompensated organ failure, major head-and-neck reconstruction within the preceding six months, inability to complete patient-reported outcome assessments, or an incomplete preoperative laboratory dataset. Patients treated with recent (chemo)radiotherapy were included only if at least six weeks had elapsed without an active inflammatory event.

### 2.2. Participants, Surgical Pathway, and Phenotype Definition

The cohort comprised 84 consecutive adults who underwent major head-and-neck oncologic reconstruction during the study period. Patients were classified into three preoperative phenotypes using three pre-specified variables obtained during the preoperative work-up: the modified five-item frailty index (mFI-5), the C-reactive protein to albumin ratio (CAR), and serum albumin. Allocation followed mutually exclusive, hierarchically applied criteria. The low-risk phenotype required an mFI-5 of 0 or 1, CAR below 1.0, and albumin of at least 3.8 g/dL. The combined frailty-inflammatory phenotype required an mFI-5 of 2 or higher together with CAR of at least 1.5 and/or albumin below 3.4 g/dL. The intermediate phenotype comprised the remaining patients, who met partial criteria of vulnerability. Cut-points were taken from published mFI-5 thresholds in head-and-neck surgery [6,7], CAR thresholds reported in oncologic outcome studies [9,13], and the conventional clinical threshold for hypoalbuminemia [11,12]. Allocation was performed independently by two investigators blinded to outcomes, with disagreements adjudicated by a senior reconstructive surgeon. Group sizes were 28, 29, and 27.

All procedures were performed by the same multidisciplinary team, minimizing operator-related variability. Postoperative care followed a standardized institutional Enhanced Recovery After Surgery (ERAS) protocol with uniform antibiotic prophylaxis, structured flap monitoring, early nutritional support, and protocolized swallowing rehabilitation. All patients underwent preoperative dental and otorhinolaryngologic screening; those with active infection or mucositis were optimized and rescheduled rather than excluded.

Patients received free-flap or pedicled-flap reconstruction according to defect complexity and operative strategy. Perioperative variables comprised operative time, ICU stay, total length of stay, PEG and tracheostomy status at discharge, any 30-day complication, major complications (Clavien–Dindo grade III or greater), and unplanned reoperation within 14 days. We hypothesized that the combined phenotype would show longer hospitalization, a greater complication burden, and poorer recovery.

### 2.3. Outcomes and Follow-Up Framework

Twelve-week outcomes captured both functional recovery and patient experience: percentage weight loss from baseline, FOIS as the graded swallowing endpoint, and PEG dependence as a binary indicator of nutritional dependency. Tracheostomy dependence was tracked separately because persistent airway support may not move in parallel with oral intake. A composite poor-recovery endpoint was defined as PEG dependence, FOIS of 3 or less, or EORTC global health below 50 at 12 weeks.

Patient-reported outcomes used complementary validated instruments: MDADI (dysphagia-specific quality of life), EORTC global health, the H&N35 swallowing domain (symptom burden), and HADS-Anxiety and HADS-Depression. Baseline and 12-week paired values were retained for the principal measures so that both final scores and within-patient change could be examined.

Two exploratory analytic layers were pre-planned. Compact multivariable logistic and linear models estimated adjusted associations for poor recovery and 12-week MDADI, respectively. Reconstruction-stratified subgroup comparisons and correlation analyses were also performed; because they involve small strata and unadjusted estimates, they are reported as Supplementary Tables S1 and S2 and are treated as descriptive.

An unsupervised recovery-cluster analysis used five 12-week outcomes (FOIS, MDADI, global health, swallowing burden, and weight loss) to illustrate how recovery states may coalesce into favorable, intermediate, and stalled patterns. This step is exploratory and is not intended as validation of the phenotype definition.

### 2.4. Statistical Analysis

Continuous variables are summarized as mean ± standard deviation and categorical variables as number and percentage. Between-group differences were assessed by one-way analysis of variance for continuous variables and by chi-square tests for categorical variables. Paired baseline-to-12-week comparisons used paired *t*-tests within each phenotype. Two-sided *p*-values below 0.05 were considered statistically significant. Because the cohort comprises only 84 patients distributed across three groups, the precision of every estimate is inherently limited. All descriptive statistics, effect estimates, and confidence intervals are therefore reported to no more than two significant figures, and *p*-values are given to two decimal places (to three decimals when below 0.01, and as *p* < 0.001 when smaller). No formal sample-size or power calculation was performed, and the study was not powered to detect small between-group differences.

Binary logistic regression estimated adjusted odds ratios for the composite poor-recovery endpoint, and ordinary least-squares regression estimated adjusted beta coefficients for 12-week MDADI; each model was deliberately restricted to four or five predictors. With 38 composite events the logistic model operates at fewer than ten events per variable, so its coefficients and confidence intervals are unstable. It is therefore presented as exploratory rather than as a validated prediction rule, and no internal validation, calibration assessment, or variable-selection procedure was undertaken. Correlation analyses used Pearson coefficients, and recovery clusters were derived by k-means from standardized 12-week outcomes.

Additional acute-phase markers—fibrinogen, procalcitonin, ferritin, and the total and differential leukocyte count—were considered but were not uniformly available at the preoperative work-up, and their sampling timing was heterogeneous. Only CRP, serum albumin, and the derived CRP/albumin ratio were therefore retained for phenotype allocation; the implications are addressed in the Limitations.

The authors used ChatGPT (GPT-5), an AI language model developed by OpenAI (San Francisco, CA, USA), to exclusively improve the manuscript’s language and readability.

## 3. Results

A total of 84 patients were analyzed: 28 low-risk, 29 intermediate, and 27 combined frailty-inflammatory. Free-flap reconstruction was used in 52 patients (62%) and pedicled reconstruction in 32 (38%). In line with the sample size, all estimates below are rounded to two significant figures.

Table 1 shows graded biologic differences across phenotypes. The low-risk group was the youngest (55 ± 9 years) and the combined phenotype the oldest (63 ± 9 years; *p* = 0.003). mFI-5 rose from 1.0 ± 0.7 to 3.2 ± 0.6 and the CRP/albumin ratio from 0.6 ± 0.2 to 2.2 ± 0.6, while albumin fell from 4.1 ± 0.3 to 3.3 ± 0.4 g/dL (all *p* < 0.001). Advanced-stage disease and planned radiotherapy did not differ significantly.

Table 2 shows a more difficult perioperative course with increasing phenotype burden. Operative time did not differ significantly (*p* = 0.19). ICU stay rose from 1.3 ± 0.5 to 2.1 ± 0.7 days and hospital stay from 11 ± 2 to 13 ± 2 days (both *p* < 0.001). Any 30-day complication occurred in 14% of low-risk versus 52% of combined-phenotype patients (*p* = 0.01); major complications reached 48% (*p* = 0.001) and PEG at discharge 56% (*p* = 0.02) in the combined phenotype.

**Figure 1:**
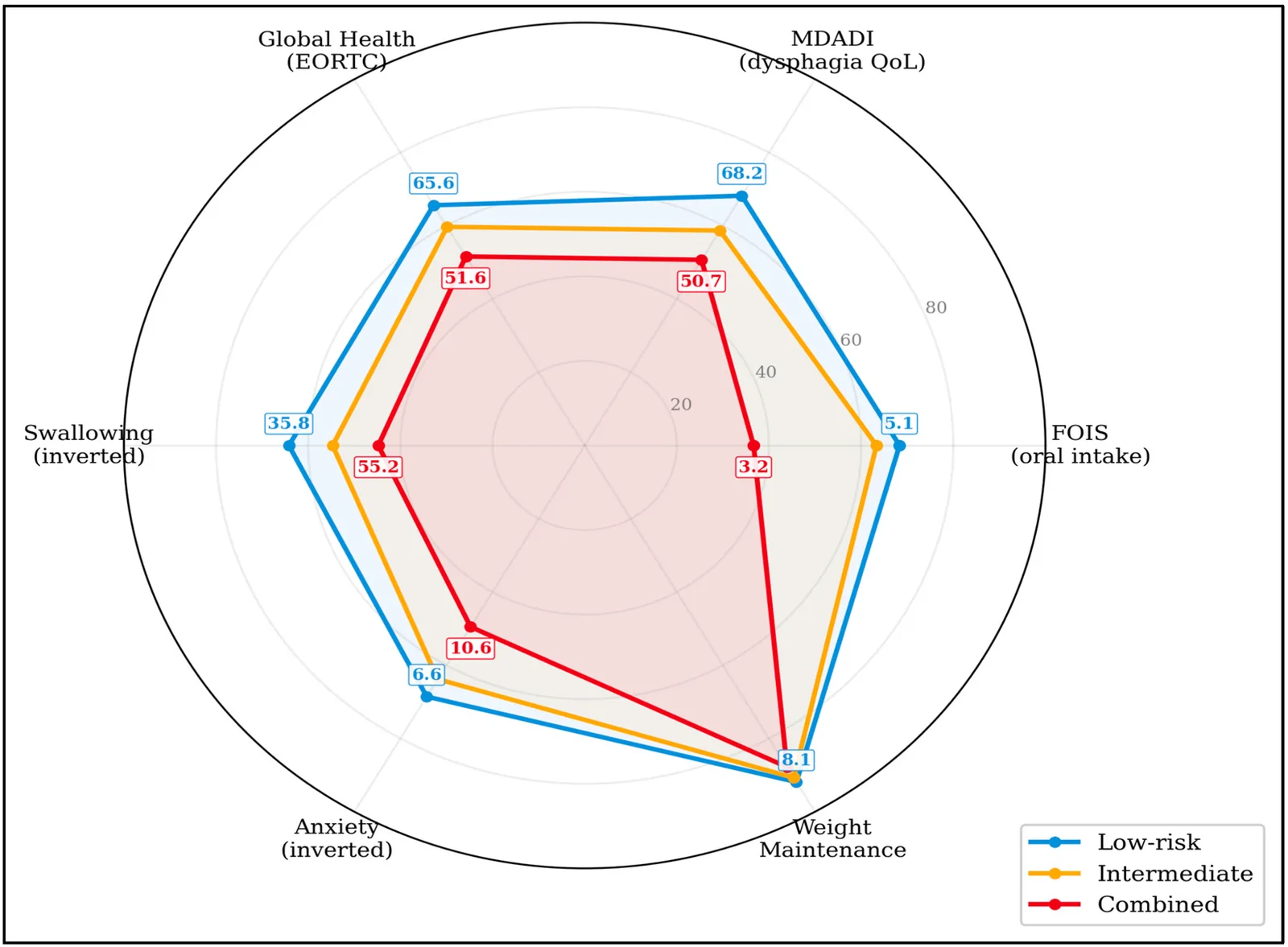
Radar profile of 12-week recovery by preoperative phenotype. All domains are scaled so that larger values indicate a more favorable state. The combined frailty-inflammatory phenotype forms the smallest polygon, indicating multidomain recovery compression. Shaded areas indicate the region enclosed by each phenotype’s polygon and share the color of the corresponding line.

**Figure 2:**
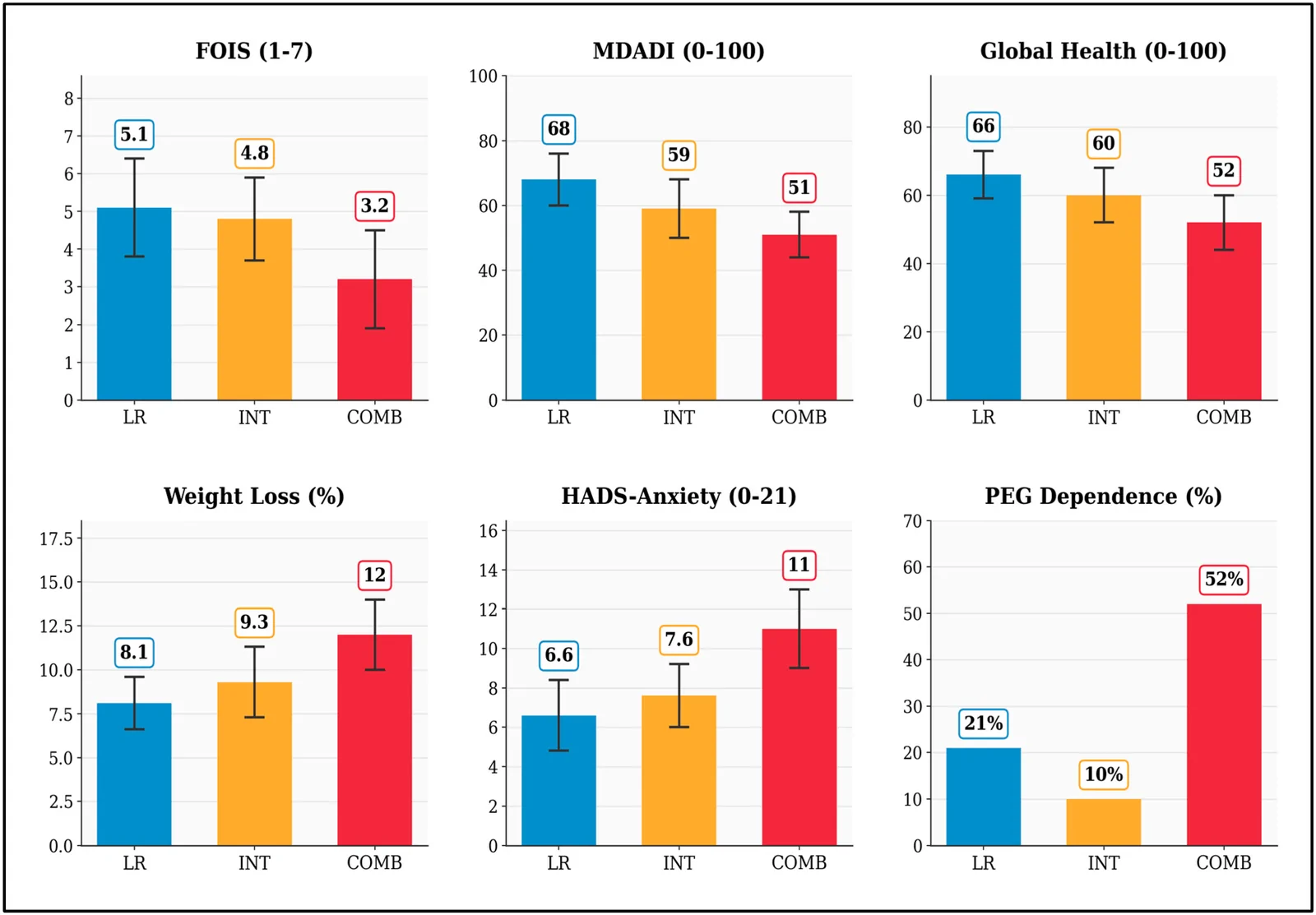
Grouped comparison of key 12-week outcomes across preoperative phenotypes. Values are rounded to two significant figures and error bars represent standard deviations. LR = low-risk; INT = intermediate; COMB = combined frailty-inflammatory phenotype.

Table 3 presents the principal outcome comparison (Figure 1). Weight loss rose from 8.1 ± 1.5% to 12 ± 2% (*p* < 0.001), and FOIS fell from 5.1 ± 1.3 to 3.2 ± 1.3 (*p* < 0.001). MDADI declined from 68 ± 8 to 51 ± 7 (*p* < 0.001; Figure 2). HADS-Anxiety and HADS-Depression reached 11 ± 2 and 9.7 ± 2.6 in the combined group (both *p* < 0.001). PEG dependence was 52% in the combined phenotype versus 21% and 10% in the other groups (*p* = 0.002), and the composite poor-recovery endpoint was met by 25 of 27 combined-phenotype patients (93%; *p* < 0.001).

Table 4 shows that the combined phenotype was not merely slower to improve: HADS-Anxiety rose by 1.3 ± 4.3 points, whereas it fell by 1.4 ± 3.2 points in the low-risk group (*p* = 0.02). HADS-Depression behaved similarly (*p* = 0.05). Because the standard deviations are large relative to the mean changes, these differences should be interpreted cautiously.

Reconstruction-stratified subgroup analyses and the full correlation matrix are provided as Supplementary Tables S1 and S2. Within-stratum numbers were as low as seven patients, and the correlations are unadjusted; both analyses are descriptive and are not used to support any inferential claim.

Exploratory unadjusted correlations linked older age with a higher mFI-5 (r = 0.46) and a higher CRP/albumin ratio (r = 0.32) and linked the CRP/albumin ratio with major complications (r = 0.37) and longer ICU stay (r = 0.41; all *p* ≤ 0.003). These variables are strongly inter-related rather than independent, and no causal ordering can be inferred from a cohort of this size.

In Table 5, mFI-5 showed the strongest independent association with poor recovery (adjusted OR 2.1 per point; 95% CI 1.0–4.1; *p* = 0.04). The confidence interval approaches unity and in-sample discrimination was good (AUC = 0.87), but with 38 events and four predictors, this estimate is imprecise and requires external validation. The corresponding model-predicted probability curves are shown in Supplementary Figure S1.

Table 6 shows that both higher-risk phenotypes were associated with lower 12-week MDADI. The adjusted R^2^ of 0.48 describes in-sample fit in 84 patients and should not be read as evidence of predictive performance.

**Figure 3:**
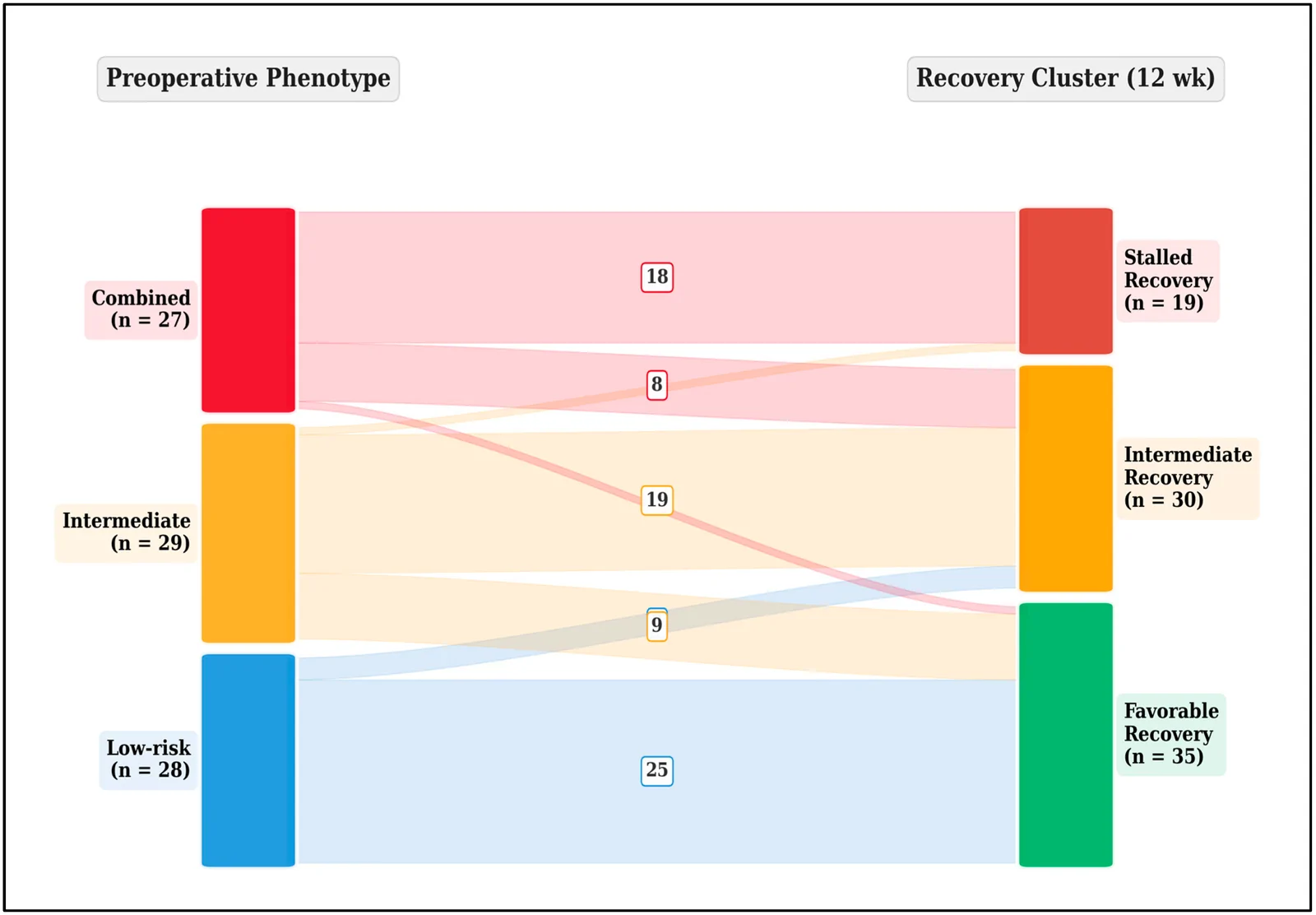
Alluvial flow diagram showing the mapping between preoperative frailty-inflammatory phenotypes and 12-week recovery clusters identified by unsupervised analysis. Flow width is proportional to the number of patients. Nearly all combined-phenotype patients transitioned into the stalled recovery cluster.

Table 7 shows that the combined phenotype accounted for 95% of the stalled recovery cluster (18 of 19 patients) and was almost absent from the favorable cluster (Figure 3). Because the clusters were derived from the same 12-week outcomes that define poor recovery, this concordance illustrates internal consistency rather than independent validation.

## 4. Discussion

### 4.1. Analysis of Findings

Early recovery after major head-and-neck reconstruction was patterned by preoperative physiologic vulnerability, and in this cohort, that vulnerability was better captured by a frailty-inflammatory phenotype than by conventional surgical descriptors. The combined phenotype entered surgery with worse reserve markers and reached 12 weeks with poorer oral intake, greater weight loss, worse dysphagia-related quality of life, and greater emotional burden—a direction of effect consistent across every instrument examined, in keeping with the multidimensional nature of recovery described previously [19,20,21].

The association between mFI-5 and poor recovery is consistent with evidence linking frailty indices to adverse surgical outcomes in head-and-neck populations [6,8]; the mFI-5 is practical because it derives from readily available comorbidity data [7]. The CRP/albumin ratio moved in the expected adverse direction without reaching significance in the logistic model [9,22], suggesting that systemic inflammation may act partly through the same pathways as frailty rather than as a fully independent risk axis [10,23].

Within the low-risk phenotype, free-flap recipients had a higher 12-week FOIS than pedicled-flap recipients (Supplementary Table S1), whereas in the combined phenotype outcomes were poor irrespective of technique [24,25]. With as few as seven patients per stratum this observation is descriptive only, although it is directionally consistent with reports from other surgical oncology settings [26,27].

The combined phenotype not only ended with worse emotional scores but showed worsening anxiety and depression from baseline to 12 weeks, while the low-risk group improved. Distress may impair engagement with swallowing rehabilitation and follow-up [21,28], and a bidirectional relationship between emotional status and functional recovery is well described in head-and-neck quality-of-life research [24,29], supporting structured psychosocial screening.

The unsupervised cluster analysis grouped patients into favorable, intermediate, and stalled recovery states that aligned with the preoperative phenotypes. Because the clustering variables overlap with the outcomes used to define poor recovery, this alignment demonstrates internal coherence rather than validation, although similar approaches may be worth pursuing prospectively [30,31].

Clinically, these observations are compatible with a shift from reactive complication management toward earlier, phenotype-guided intervention. Simple preoperative markers such as mFI-5 and the CRP/albumin ratio could flag patients for intensified nutritional optimization, earlier swallowing therapy, and closer psychosocial follow-up, consistent with ESPEN guidance on nutritional risk screening in surgical oncology [12,32,33]. Any such pathway would itself require prospective evaluation.

Elevated CRP-based inflammatory–nutritional indices have been linked with higher pathologic stage, more postoperative complications, and inferior disease-specific survival in head-and-neck squamous cell carcinoma [9,13,34]. Related composite scores—the neutrophil-to-lymphocyte ratio, the Glasgow Prognostic Score, and the prognostic nutritional index—capture overlapping dimensions [9,10]. The phenotype used here adds a structured frailty dimension and relates preoperative biology to multidimensional recovery rather than to survival alone [28,31], but whether it genuinely refines early recovery estimation beyond either marker alone cannot be established in a cohort of this size.

Local infectious factors also merit consideration. Untreated odontogenic infection, periodontal disease, and sinonasal inflammation can seed the operative field and increase the risk of wound infection, salivary fistula, and impaired flap integration [15,35]. All patients here underwent structured preoperative dental and otorhinolaryngologic screening, but residual variability in its timing and completeness remains.

### 4.2. Study Limitations

The most important limitation of this study is its size. Eighty-four patients were distributed across three phenotypes (28, 29, and 27), and the composite poor-recovery endpoint occurred in only 38 patients. Every estimate reported here is therefore imprecise: confidence intervals are wide, the subgroup and cluster analyses are underpowered, and the findings must be read as hypothesis-generating rather than as evidence of predictive validity. No sample-size calculation was performed, multiplicity was not formally controlled across the several outcomes examined, and small denominators make percentages unstable, since a single patient shifts a group proportion by roughly four percentage points. For these reasons all results are reported to two significant figures, and no clinical decision rule should be derived from them.

Further limitations follow. The prospective single-center design limits generalizability and remains susceptible to selection bias; patients judged unfit for reconstruction were never operated on, so the estimates are conditional on the decision to proceed. Combined-phenotype patients were older, more often smokers, and tended toward more advanced stage, and residual confounding by socioeconomic status, health literacy, cognitive reserve, social support, rehabilitation access, and adherence cannot be excluded [36,37,38,39,40,41,42,43,44]. The phenotype definitions were deliberately simplified and omit formal sarcopenia thresholds and performance-status measures. Follow-up was limited to 12 weeks. The inflammatory characterization relied on CRP and albumin alone, because fibrinogen, procalcitonin, ferritin, and differential leukocyte counts were not uniformly available. There was no external validation cohort. Prospective, adequately powered, multicenter studies with longer follow-up are required before this framework is used to guide care.

## 5. Conclusions

In this exploratory single-center cohort of 84 patients, a preoperative frailty-inflammatory phenotype was associated with early recovery after head-and-neck reconstruction. Patients with the combined phenotype entered surgery with lower albumin, higher CRP/albumin ratios, and lower skeletal muscle reserve, and subsequently had longer hospital stays, more major complications, greater weight loss, lower oral intake, and worse patient-reported outcomes at 12 weeks. The cohort is too small to support conclusions about predictive accuracy. The framework is best regarded as a hypothesis for prospective, adequately powered, multicenter testing.

## Figures and Tables

**Table 1:** Baseline characteristics by preoperative frailty-inflammatory phenotype.

Variable	Low-Risk Phenotype	Intermediate Phenotype	Combined Frailty-Inflammatory	*p*
Age, years	55 ± 9	59 ± 8	63 ± 9	0.003
Male sex, *n* (%)	21 (75)	22 (76)	19 (70)	0.88
BMI, kg/m^2^	28 ± 3	27 ± 4	25 ± 4	0.08
mFI-5 frailty score (0–5)	1.0 ± 0.7	1.7 ± 0.9	3.2 ± 0.6	<0.001
CRP/albumin ratio	0.6 ± 0.2	1.2 ± 0.2	2.2 ± 0.6	<0.001
Albumin, g/dL	4.1 ± 0.3	3.6 ± 0.4	3.3 ± 0.4	<0.001
Skeletal muscle index, cm^2^/m^2^	45 ± 5	41 ± 4	40 ± 4	<0.001
Advanced stage (III–IV), *n* (%)	17 (61)	21 (72)	22 (81)	0.23
Planned adjuvant radiotherapy, *n* (%)	19 (68)	23 (79)	22 (81)	0.44
Current smoker, *n* (%)	6 (21)	9 (31)	13 (48)	0.10
Baseline FOIS (1–7)	4.1 ± 1.0	4.1 ± 1.0	3.2 ± 1.1	0.003
Baseline MDADI composite (0–100)	61 ± 10	57 ± 8	51 ± 9	<0.001

Abbreviations: BMI, body mass index; FOIS, Functional Oral Intake Scale; MDADI, MD Anderson Dysphagia Inventory; mFI-5, modified five-item frailty index; CRP, C-reactive protein; CRP/albumin ratio is calculated as serum CRP (mg/L) divided by serum albumin (g/L). The mFI-5 ranges from 0 to 5, with higher scores indicating greater frailty. FOIS ranges from 1 (no oral intake) to 7 (normal diet without restriction). MDADI composite scores range from 0 to 100, with higher values indicating better dysphagia-specific quality of life. Continuous variables are expressed as mean ± standard deviation; categorical variables as *n* (%). *p*-values are derived from one-way ANOVA for continuous variables and chi-square tests for categorical variables. All values are rounded to two significant figures, consistent with the size of the cohort.

**Table 2:** Perioperative course and 30-day outcomes by phenotype.

Outcome	Low-Risk	Intermediate	Combined	*p*
Free-flap reconstruction, *n* (%)	21 (75)	15 (52)	16 (59)	0.18
Operative time, hours	7.1 ± 1.3	7.2 ± 1.2	7.7 ± 1.1	0.19
ICU stay, days	1.3 ± 0.5	1.8 ± 0.6	2.1 ± 0.7	<0.001
Length of stay, days	11 ± 2	12 ± 2	13 ± 2	<0.001
PEG at discharge, *n* (%)	7 (25)	7 (24)	15 (56)	0.02
Tracheostomy at discharge, *n* (%)	7 (25)	9 (31)	11 (41)	0.45
Any 30-day complication, *n* (%)	4 (14)	8 (28)	14 (52)	0.01
Major complication (Clavien–Dindo ≥ III), *n* (%)	4 (14)	3 (10)	13 (48)	0.001
Unplanned reoperation ≤ 14 days, *n* (%)	3 (11)	3 (10)	4 (15)	0.85

Abbreviations: ICU, intensive care unit; PEG, percutaneous endoscopic gastrostomy. Clavien–Dindo grade refers to the standardized classification of postoperative complications; grade III or greater indicates the need for surgical, endoscopic, or radiologic intervention, ICU management, or organ failure. Unplanned reoperation includes any return to the operating room within 14 days of the index procedure for indications other than pre-planned staged reconstruction. Continuous variables are reported as mean ± standard deviation; categorical variables as *n* (%).

**Table 3:** Twelve-week functional, nutritional, and patient-reported outcomes by phenotype.

Outcome	Low-Risk	Intermediate	Combined	*p*
Weight loss from baseline, %	8.1 ± 1.5	9.3 ± 2.0	12 ± 2	<0.001
FOIS at 12 weeks (1–7)	5.1 ± 1.3	4.8 ± 1.1	3.2 ± 1.3	<0.001
MDADI at 12 weeks (0–100)	68 ± 8	59 ± 9	51 ± 7	<0.001
EORTC global health at 12 weeks (0–100)	66 ± 7	60 ± 8	52 ± 8	<0.001
EORTC H&N35 swallowing at 12 weeks (0–100; higher = worse)	36 ± 8	45 ± 8	55 ± 10	<0.001
HADS-Anxiety at 12 weeks (0–21)	6.6 ± 1.8	7.6 ± 1.6	11 ± 2	<0.001
HADS-Depression at 12 weeks (0–21)	5.6 ± 2.1	7.3 ± 2.0	9.7 ± 2.6	<0.001
PEG dependence at 12 weeks, *n* (%)	6 (21)	3 (10)	14 (52)	0.002
Tracheostomy dependence at 12 weeks, *n* (%)	2 (7)	4 (14)	4 (15)	0.63
Poor recovery composite, *n* (%)	7 (25)	6 (21)	25 (93)	<0.001

Abbreviations: EORTC, European Organisation for Research and Treatment of Cancer; FOIS, Functional Oral Intake Scale; HADS, Hospital Anxiety and Depression Scale; PEG, percutaneous endoscopic gastrostomy; MDADI, MD Anderson Dysphagia Inventory; H&N35, EORTC Head and Neck cancer module (35 items); higher EORTC global health scores indicate better health-related quality of life, whereas higher H&N35 swallowing scores indicate worse symptom burden. HADS-Anxiety and HADS-Depression each range from 0 to 21, with higher scores indicating greater symptom severity. The poor-recovery composite endpoint was defined as PEG dependence, FOIS ≤ 3, or EORTC global health below 50 at 12 weeks. *p*-values were derived from one-way ANOVA for continuous variables and chi-square tests for categorical variables.

**Table 4:** Patient-reported outcome trajectories from baseline to 12 weeks.

Measure	Low-Risk Δ	Intermediate Δ	Combined Δ	*p* Δ Difference
MDADI composite	+6.9 ± 11	+2.0 ± 11	−0.2 ± 12	0.07
EORTC global health	+8.6 ± 11	+4.1 ± 13	+2.6 ± 11	0.13
EORTC H&N35 swallowing	−8.2 ± 12	−3.8 ± 13	−5.8 ± 13	0.44
HADS-Anxiety	−1.4 ± 3.2	−0.8 ± 3.3	+1.3 ± 4.3	0.02
HADS-Depression	−2.0 ± 3.7	−1.3 ± 4.3	+0.6 ± 4.0	0.05

Δ indicates change from 12 weeks minus baseline. Positive Δ is better for MDADI/global health; negative Δ is better for swallowing burden/HADS. Abbreviations: MDADI, MD Anderson Dysphagia Inventory; EORTC, European Organisation for Research and Treatment of Cancer; H&N35, EORTC Head and Neck cancer module; HADS, Hospital Anxiety and Depression Scale. The reported *p*-value reflects between-group comparison of within-patient paired changes using one-way ANOVA; all changes are within-patient differences computed at the individual level before group aggregation. Values are mean ± standard deviation of the within-patient change.

**Table 5:** Multivariable logistic regression for the composite poor-recovery endpoint (exploratory).

Predictor	aOR (95% CI)	*p*
mFI-5 (per point)	2.1 (1.0–4.1)	0.04
CRP/albumin ratio (per unit)	2.5 (0.81–7.5)	0.11
Baseline FOIS (per point)	0.59 (0.34–1.0)	0.07
Major complication (yes vs. no)	1.5 (0.35–6.7)	0.57

Model discrimination: AUC = 0.87. Abbreviations: aOR, adjusted odds ratio; CI, confidence interval; AUC, area under the receiver operating characteristic curve; mFI-5, modified five-item frailty index; CRP, C-reactive protein; FOIS, Functional Oral Intake Scale. The composite poor-recovery endpoint was defined as PEG dependence, FOIS ≤ 3, or EORTC global health below 50 at 12 weeks. The model was estimated by binary logistic regression including all four predictors simultaneously; *p*-values are two-sided. Estimates derive from 38 events and four predictors (fewer than ten events per variable), were not internally or externally validated, and are exploratory; the AUC reflects apparent (in-sample) discrimination.

**Table 6:** Multivariable linear regression for 12-week MDADI (exploratory).

Predictor	β (95% CI)	*p*
Intermediate phenotype vs. low-risk	−8.4 (−13 to −4.1)	<0.001
Combined phenotype vs. low-risk	−12 (−18 to −5.9)	<0.001
Major complication (yes vs. no)	−4.8 (−9.0 to −0.47)	0.03
Weight loss (per 1%)	−0.79 (−1.7 to 0.15)	0.10
Baseline MDADI (per point)	0.07 (−0.12 to 0.26)	0.44

Adjusted R^2^ = 0.48. Negative beta values indicate lower MDADI at 12 weeks. Abbreviations: β, regression beta coefficient (estimated change in 12-week MDADI per one-unit change in predictor or relative to the reference category); CI, confidence interval; MDADI, MD Anderson Dysphagia Inventory. The reference category for phenotype was the low-risk phenotype; the reference category for major complication was the absence of a Clavien–Dindo ≥ III event. The model was estimated by ordinary least-squares regression including all listed predictors simultaneously. The adjusted R^2^ describes in-sample fit in 84 patients and is not a measure of predictive accuracy.

**Table 7:** Recovery-cluster profiling based on 12-week outcomes (exploratory).

Recovery Cluster	*n*	FOIS 12w	MDADI 12w	Global Health	Weight Loss %	PEG 12w	Major Comp	Phenotype Mix L/I/C (%)
Favorable recovery	35	4.9 ± 1.3	69 ± 7	65 ± 7	8.1 ± 1.5	8 (23%)	5 (14%)	71/26/3
Intermediate recovery	30	4.9 ± 0.9	54 ± 6	58 ± 7	10 ± 2	9 (30%)	4 (13%)	10/63/27
Stalled recovery	19	2.6 ± 0.9	50 ± 7	49 ± 7	13 ± 1	6 (32%)	11 (58%)	0/5/95

Phenotype mix is shown as Low-risk/Intermediate/Combined percentages within each recovery cluster. Abbreviations: FOIS 12w, Functional Oral Intake Scale at 12 weeks; MDADI 12w, MD Anderson Dysphagia Inventory composite score at 12 weeks; Global health, EORTC QLQ-C30 global health/quality-of-life subscale at 12 weeks; PEG 12w, percutaneous endoscopic gastrostomy dependence at 12 weeks; Major comp, major complication defined as a Clavien–Dindo grade III or higher event during the index hospitalization; L/I/C, Low-risk/Intermediate/Combined frailty-inflammatory phenotype. Clusters were derived from unsupervised k-means analysis using standardized 12-week functional and patient-reported features. Clusters were derived from the same 12-week outcomes that define the composite poor-recovery endpoint; they are descriptive and do not constitute independent validation.

## Data Availability

The data presented in this study are available on request from the corresponding author due to privacy and ethical reasons.

## Supplementary Materials

The following supporting information can be downloaded at: https://www.mdpi.com/article/10.3390/jcm15187134/s1, Table S1: Reconstruction-stratified subgroup analysis within each phenotype; Table S2: Correlations linking physiologic vulnerability and multidimensional recovery; Figure S1: Model-predicted probability of poor recovery according to CRP/albumin ratio across frailty scenarios. Curves represent predicted probabilities from the exploratory logistic model (Table 5 of the main text) with baseline FOIS held at 4 and no major complication; overlaid markers show observed phenotype-level poor-recovery rates. The model was fitted to 84 patients with 38 composite events and four predictors, was not internally or externally validated, and the curves extend across a range of CRP/albumin values that is sparsely populated in this cohort. The figure is illustrative only and must not be used as a clinical prediction tool.
