# Supplementary material for: Preoperative Frailty-Inflammation Phenotypes and Early Functional and Patient-Reported Recovery After Head-and-Neck Oncologic Reconstruction: An Exploratory Prospective Single-Center Cohort Study
Source: J Clin Med. 2026 Sep 14;15(18):7134. doi: 10.3390/jcm15187134 (PMC13607148; doi:10.3390/jcm15187134)
Supplement: Supplementary file 1 [file jcm-15-07134-s001.zip › jcm-4295812-supplementary.pdf]

Supplementary Materials

Preoperative Frailty-Inflammation Phenotypes and Early Functional and Patient-Reported Recovery After Head-and-Neck Oncologic Reconstruction: An Exploratory Single-Center Cohort Study

Manuscript ID: jcm-4295812

The analyses below were relocated from the main text during revision. All are exploratory, involve small strata or unadjusted estimates, and are reported to two significant figures. They are provided for completeness and are not used to support any inferential claim in the manuscript.

Table S1. Reconstruction-stratified subgroup analysis within each phenotype.

Phenotype	n Free	n Pedicled	FOIS Free	FOIS Pedicled	p FOIS	PEG Free	PEG Pedicled	p PEG
Low-risk	21	7	5.4 ± 1.2	4.1 ± 0.7	0.003	3 (14%)	3 (43%)	0.29
Intermediate	15	14	4.8 ± 1.2	4.7 ± 1.0	0.83	1 (6.7%)	2 (14%)	0.95
Combined	16	11	3.5 ± 1.1	2.9 ± 1.6	0.32	8 (50%)	6 (55%)	1.00

Abbreviations: Free, microvascular free flap reconstruction; Pedicled, pedicled regional flap reconstruction; FOIS, Functional Oral Intake Scale (mean ± standard deviation); PEG, percutaneous endoscopic gastrostomy dependence at 12 weeks. p-values compare free-flap and pedicled-flap recipients within each phenotype stratum using Welch's t-test for FOIS and Fisher's exact test for PEG dependence. Within-stratum sample sizes are as small as seven patients; these comparisons are descriptive and should not be interpreted inferentially.

Table S2. Correlations linking physiologic vulnerability and multidimensional recovery.

Variable 1	Variable 2	r	p
MDADI at 12 weeks	Global health at 12 weeks	0.41	<0.001
MDADI at 12 weeks	H&N35 swallowing at 12 weeks	−0.53	<0.001
FOIS change	MDADI at 12 weeks	0.44	<0.001
Weight loss (%)	Global health at 12 weeks	−0.48	<0.001
CRP/albumin ratio	FOIS at 12 weeks	−0.51	<0.001
mFI-5	Poor recovery (binary)	0.56	<0.001

Pearson correlation coefficients are shown; positive r indicates a direct association and negative r an inverse association. Abbreviations: MDADI, MD Anderson Dysphagia Inventory; FOIS, Functional Oral Intake Scale; H&N35, EORTC Head and Neck cancer module (swallowing domain); CRP, C-reactive protein; mFI-5, modified five-item frailty index; Poor recovery (binary), composite endpoint defined as PEG dependence, FOIS ≤ 3, or EORTC global health below 50 at 12 weeks. All correlations are unadjusted, are computed in 84 patients, and cannot establish causal ordering. p-values are two-sided.

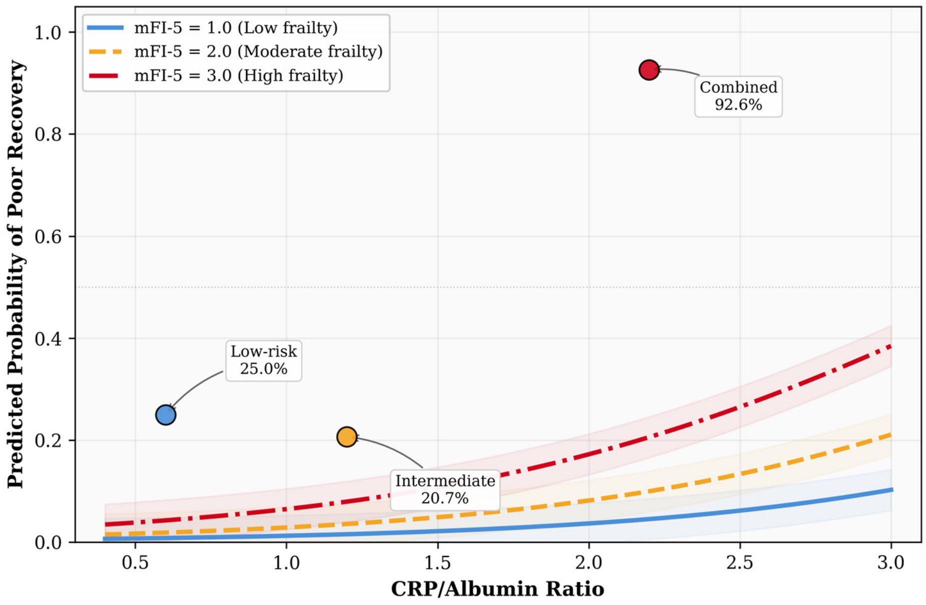

Figure S1. Model-predicted probability of poor recovery according to CRP/albumin ratio across frailty scenarios. Curves represent predicted probabilities from the exploratory logistic model (Table 5 of the main text) with baseline FOIS held at 4 and no major complication; overlaid markers show observed phenotype-level poor-recovery rates. The model was fitted to 84 patients with 38 composite events and four predictors, was not internally or externally validated, and the curves extend across a range of CRP/albumin values that is sparsely populated in this cohort. The figure is illustrative only and must not be used as a clinical prediction tool.
